# Assessing the Knowledge and Beliefs of Greek Dietitians and Nutritionists on Nutritional Genomics: A Survey-Based Study

**DOI:** 10.3390/nu17071107

**Published:** 2025-03-21

**Authors:** Ioanna Panagiota Kalafati, Theodora Alexandrou, Evangelia Mentsiou-Nikolaou, Michael Chourdakis, George V. Dedoussis

**Affiliations:** 1 Department of Nutrition and Dietetics, School of Physical Education, Sport Science and Dietetics, University of Thessaly, 42132 Trikala, Greece; ikalafati@uth.gr; 2 Department of Nutrition and Dietetics, School of Health Science and Education, Harokopio University, 17671 Athens, Greece; evemen@hua.gr; 3 School of Medicine, Faculty of Health Sciences, Aristotle University of Thessaloniki, 54124 Thessaloniki, Greece; dora.alexandrou19@gmail.com (T.A.); mhourd@gapps.auth.gr (M.C.)

**Keywords:** dietitians, nutritionists, nutrigenetics, nutrigenomics, nutritional genomics, knowledge

## Abstract

**Background/Objectives**: The field of Nutritional Genomics represents a growing area of interest among dietitians and nutritionists. However, knowledge gaps persist globally, limiting the effective application of this science. This study aims to evaluate the demographic characteristics, knowledge levels, and perspectives of Greek dietitians and nutritionists regarding Nutritional Genomics. **Methods**: A survey was conducted among 155 Greek dietitians and nutritionists from diverse educational and professional backgrounds. A 25-item questionnaire was designed to assess knowledge in Nutritional Genomics, with reliability measured by Cronbach’s Alpha (α = 0.867). Statistical analyses, including chi-square tests and regression modeling, were employed to explore associations between knowledge scores [S (total), E (easy questions), and H (hard questions) scores] and demographic or professional factors. Participants’ views on the clinical utility and future implications of Nutritional Genomics were also assessed. **Results**: On average, participants replied correctly to 57.7% (±21.7%) of the questions, with significant differences observed based on education level, employment status, and prior interest in genetics. Women demonstrated higher S and E scores compared to men (*p* = 0.046 and *p* = 0.006, respectively), while younger participants (26–35 years) outperformed those over 45 years (*p* = 0.019). Despite moderate knowledge levels, 76.8% believed Nutritional Genomics could benefit their clients, and 77.4% expressed interest in specializing in this field. **Conclusions**: Greek dietitians and nutritionists exhibited moderate knowledge in Nutritional Genomics, similar to international findings. Education level, age, and prior exposure to genetics significantly influenced knowledge scores. These results underscore the need for the integration of Nutritional Genomics into dietetic curricula and continuing professional education.

## 1. Introduction

Today, the immense evolution in the field of genetics, specifically after the decoding of the human genome, has greatly influenced the field of nutrition [1]. Potential associations and interactions between single nucleotide polymorphisms (SNPs) and dietary factors (nutrients or specific dietary patterns) are now being extensively investigated, particularly considering their role in the expression of certain phenotypes or the predisposition to common multifactorial diseases [2]. The term Nutritional Genomics, which mainly refers to the fields of nutrigenomics and nutrigenetics [3], has been developed since the 1990s [4] in order to describe the functional interplay between food and the genome at the cellular and at the molecular level, aiming to make nutrition a tool for the prevention and treatment of diseases [5]. Specifically, the field of nutrigenomics examines how nutrients influence gene expression [6]. This area of research investigates the entire set of genes that may be positively or negatively altered by dietary factors, such as the Mediterranean diet and specific nutrients (trace elements, vitamins, fatty acids). The field of nutrigenetics explores how SNPs in genes influence an individual’s response to food components or dietary interventions [7,8]. It should be stated that identifying the “specific traits” of each organism is important for scientists to evaluate both their positive or negative impact on the population’s dietary habits and vice versa.

Due to technological and scientific advances, nutritional research has now moved into a new era of personalized nutrition. Among other aspects, personalized nutrition involves the use of genetic tests to provide tailored dietary recommendations based on an individual’s genotype, aiming to prevent disease or improve one’s health in a more efficient way [9]. Nowadays, nutrigenetic tests are used by trained dietitians and nutritionists to determine each individual’s specific needs for macronutrients (proteins, carbohydrates, fats), micronutrients (such as vitamins, minerals and trace elements), alcohol, caffeine, and salt, thus embracing tailor-made recommendations for each case. These tests identify SNPs linked to a predisposition for diseases or disorders (such as hypertension, obesity, and cardiovascular diseases) or food-related intolerances (like lactose or gluten). However, the interpretation of the tests’ results and the application of this knowledge are still in the early stages. It has been proposed that genetic tests should be evaluated based on four criteria: analytical validity, clinical validity, clinical utility, and the associated ethical, legal, and social implications [10,11].

Many laboratories and companies, in collaboration with healthcare professionals (HCPs), use genetic tests, known as Direct-to-Consumer Genetic Testing (DTCGT), to promote health, encourage healthy dietary habits among individuals, and support personalized nutrition [11]. Despite their advantages, both genetic tests and the information provided by the relevant companies carry some risks. According to a survey, the number of companies that provide nutrigenetic testing increased significantly between 2013 and 2015 [12]. That same year, the American Society of Human Genetics recommended deterring professionals from using DTCGT on children until companies could ensure the quality, accuracy, and validity of the tests, and also ensure that there is adequate post-test counseling. In Greece, regulations such as the General Data Protection Regulation (GDPR) 2016/679, which is an EU regulation governing privacy, apply to food, dietary supplements, medicines, and pharmaceutical products in medical testing, and thus apply to genetic tests conducted in laboratories. Some of these tests are overseen by the Food and Drug Administration (FDA), which ensures that they can reliably and accurately measure what they claim and whether the measurement is predictive of a specific health condition. The primary goal of the legislative framework for genetic testing is to protect individuals from any physical, emotional, or psychological harm and to safeguard their rights [13].

Recently, there has been growing interest among HCPs in using genetic tests in their daily practice. However, concerns persist about whether they have the necessary expertise to evaluate the results and whether they are accurately trained. Registered dietitians and nutritionists (RDNs) need both a base knowledge of genetics to understand the complexity of Nutritional Genomics before integrating this tool into their routine practice and to provide post-test counseling. They should also be trained through relevant courses, certifications, or graduate programs in genetics [14]. In recent years, there has been significant interest in the investigation of the theoretical knowledge of HCPs, and especially dietitians and nutritionists, on issues related to genetic testing and counseling as well as its application in practice. The aim of the present study was to assess, for the first time, the knowledge of Greek dietitians and nutritionists in relation to the science of nutrigenetics–nutrigenomics and its application in clinical practice through a questionnaire, created for this purpose by expert nutritionists, dietitians, and molecular biologists in this field. In Greece, dietitians and nutritionists must obtain a bachelor’s degree (BSc) in Nutrition and Dietetics from either a Technological Educational Institute (TEI) or a Higher Education Institution (HEI). Many pursue a master’s degree (MSc) for specialization, while a Doctor of Philosophy (PhD) is research-focused and prepares them for academic or policy-making roles. To our knowledge, there are relatively few publications on this topic in total, which makes this issue highly intriguing for the scientific community.

## 2. Materials and Methods

### 2.1. Sample Population

In the context of this survey-based study, dietitians and nutritionists all over Greece were invited to participate in the study by filling out the provided online questionnaire. To determine the sample size of the study, a literature review of similar studies was conducted both in Greece and internationally. Due to the limited number of relevant publications in scientific databases such as PubMed and Google Scholar, a minimum sample size of 100 individuals was established for this study. Subjects recruited were over 21 years old and had completed their studies in Dietetics and Nutrition in universities within Greece or abroad. No other healthcare professionals were included in the study. The final study sample consisted of 155 dietitians and nutritionists living in various places around Greece. All participants were informed about the details of the study and signed a consent form prior to their recruitment. The study and the questionnaire used herein were approved by the Research Ethics Committee of the Aristotle University of Thessaloniki in Greece (3.684/18 January 2022). All the collected data were anonymous and the EU GDPR rules were followed. No language that is stigmatizing or prejudiced will be used when referring to study participants.

### 2.2. Questionnaire

Herein, the tool chosen to collect the data was a questionnaire which was electronically set up using Google forms. Questionnaires are a very popular means of low-cost data collection and constitute a communication link between the researcher and the respondents. The questionnaire was distributed to dietitians and nutritionists all over Greece via social networking platforms (i.e., Facebook, Instagram, LinkedIn), and also forwarded via dietetic associations to their registered members. Overall, the questionnaire consisted of 42 closed-ended, dichotomous or multiple-choice questions with one possible answer. To incorporate answers not provided in the questionnaire, the residual category “other” was included. Moreover, the “I do not know” option was included in all the knowledge-related questions. To complete the questionnaire, approximately 10 min were needed, and the participants were encouraged to share their honest opinions.

The questionnaire consisted of 3 parts: the first part included demographic data-related questions (9 questions regarding sex, age, education, income, living area, professional status, and experience), the second part questions assessed the subjects’ knowledge regarding the science of Nutritional Genomics (consisting of 25 questions), and the third part evaluated the individual’s personal perspective on the adequacy of their knowledge and the practical relevance of this field through 8 targeted questions. This questionnaire was newly developed by experts in the fields of nutrition, dietetics, and molecular biology, tailored specifically for assessing the knowledge of Nutritional Genomics among dietitians. To ensure reliability, internal consistency was measured using Cronbach’s Alpha, which yielded a high reliability score of 0.867. While the survey was not previously validated through external studies, this reliability assessment supports the robustness of the questionnaire in evaluating the intended constructs. The knowledge-related questions (part two of the questionnaire—Supplementary Materials) were designed to examine the following aspects: (i) the study of SNPs, (ii) the science of nutrigenetics, (iii) the science of nutrigenomics, (iv) the information provided by nutrigenetic tests, (v) information about the purpose and process of nutrigenetic tests, (vi) the sources of information regarding this field, (vii) the process of carrying out these tests, as well as (viii) specific examples of interactions between genetic and nutritional factors or examples of specific SNPs and health problems. Within part two, individuals were also asked about their personal opinion on whether they considered their knowledge to be sufficient or useful, and on whether they believed that this field could be helpful for dietitians and nutritionists in terms of professional development. In order to assess each subject’s knowledge, a scoring system was developed. The questions in part 2 were further divided into two blocks, based on whether the questions were considered to be easy or hard. There were 25 questions in total, out of which 13 were scored as easy and 12 as hard. Questions on the general concepts of the sciences of genetics, nutrigenetics, and nutrigenomics, as well as practical questions on the use of nutrigenetic tests, were considered as easy questions. Questions on specialized knowledge in the field of Nutritional Genomics were deemed as hard. Answering a question correctly was scored as “1”, while a “0” was assigned to wrong answers. A new variable, herein called S-score, was calculated by summing the correct answers in the total number of answers. S-score ranged from 0 to 25. Similarly, E-score and H-score were created in order to reflect the number of correct answers to the easy and hard questions. Questions 1, 4, 5, 6, 8, 12, 13, 14, 21, 22, 23, 24, and 25 were considered as easy (range: 0–13), while questions 2, 3, 7, 9, 10, 11, 15, 16, 17, 18, 19, and 20 were considered hard (range: 0–12). Each person was attributed a score for all three of the scores.

### 2.3. Statistical Analysis

To evaluate the normality of the data, the Shapiro–Wilk test was employed in conjunction with graphical representation using QQ plots. All the scores were normally distributed and therefore expressed as means ± standard deviation (mean ± sd), while categorical variables were expressed as absolutes and relative frequency [*n* (%)]. To assess the reliability of the scale, Cronbach’s Alpha coefficient was analyzed, which evaluates the level of measurement error in a test. Cronbach’s Alpha values greater than or close to 0.7 (70%) are considered acceptable, while values exceeding 0.8 (80%), indicate an exceptionally high level of reliability. In order to compare the mean values of each variable among groups, independent samples *t*-tests were used for two independent variables. To study the relationship between a continuous variable and a categorical variable with three or more categories, the analysis of variance (ANOVA) test was used. A post hoc Bonferroni analysis was applied to examine subgroup comparisons individually. To assess the independence of two or more categorical variables, the chi-square test of independence was applied. A backward linear regression model was applied to assess the effect of different factors on S-score. All variables deriving from the first and third parts of the questionnaire were included in the regression model to assess their effect on the knowledge level of the dietitians/nutritionists; however, the final model contained only the most significant predictors. No covariates were added in the regression model. In all tests, the level of significance (α) was set to 0.05. Data were analyzed by using SPSS Statistics for Mac version 24.0 (Armonk, NY, USA: IBM Corp) and R version 4.4.1.

## 3. Results

### 3.1. Demographic Characteristics of Sample Population

This study included 155 dietitians and nutritionists from diverse fields and educational backgrounds. The demographic characteristics of the sample population are detailed in Table 1. Precisely, the sample consisted of 155 individuals, with 83.2% being women and 76.2% aged between 26 and 45 years. Regarding education, 33.5% of the participants were TEI graduates, 29% were HEI graduates, and 36.4% held MSc or PhD degrees. In addition, most of the participants in this study (88.4%) were employed, primarily in private dietetic offices (49%), with 68.4% having less than 10 years of experience. Regarding monthly income, most of the dietitians and nutritionists (60.6%) reported a monthly income of more than 1000 euros. Greece’s statutory minimum wage is set at €830 per month for white-collar workers (Ministry of Labour and Social Security, Hellenic Republic), which means that almost half of the dietitians/nutritionists in Greece earn monthly more than the country’s minimum wage.

The internal consistency coefficient, Cronbach’s Alpha, for the 25-question scale on Nutritional Genomics was calculated to be 0.867, which indicates a particularly high level of reliability. This result suggests that the questionnaire exhibits strong internal consistency, reinforcing its suitability for assessing knowledge levels in this domain. The reliability assessment further supports the credibility of the collected data, despite the questionnaire being newly developed. In the table below (Table 2), the questionnaire along with the percentage of respondents who answered correctly in each question are presented. On average, participants replied correctly to 57.7% (±21.7%) of the questions. One third of the participants demonstrated low knowledge of Nutritional Genomics (mean S-score = 8.06), one third demonstrated medium knowledge (mean S-score = 15.04), and the remaining third demonstrated high knowledge (mean S-score = 19.58).

Subsequently, a chi-square test was performed to examine the relationship between participants’ educational level, employment status, previous interest in genetics, and their responses to the questionnaire items. Specifically, participants’ responses to question 6 (*p*-value = 0.004), question 8 (*p*-value = 0.009), question 10 (*p*-value = 0.013), and question 18 (*p*-value = 0.005) were found to be significantly influenced by their educational level. Additionally, their responses to question 3 (*p*-value = 0.034), question 6 (*p*-value = 0.048), question 12 (*p*-value = 0.039), question 19 (*p*-value = 0.032), question 21 (*p*-value = 0.016), and question 23 (*p*-value = 0.022) were found to be significantly dependent on the participants’ employment status. Furthermore, participants’ previous interest in the field of genetics and gene–nutrition interactions was found to have a statistically significant and positive effect on replying correctly to almost all questionnaire items (*p*-value < 0.05).

### 3.2. Assessment of Knowledge on Nutritional Genomics

As shown in Table 3, women presented significantly higher knowledge in the field compared to men, as indicated by their S-scores (14.84 ± 5.19 vs. 12.15 ± 6.17, *p*-value = 0.046) and their E-scores (9.15 ± 2.81 vs. 7.38 ± 3.62, *p*-value = 0.006) on the questionnaire items. While women also scored higher in H-scores, this difference was smaller and not significant (5.69 ± 2.75 vs. 4.77 ± 3.00, *p*-value = 0.157). There was also a statistically significant difference regarding the average S-score, E-score, and H-score among age-group categories. Specifically, people aged 25–35 presented higher knowledge scores compared to people aged over 45 years, whereas no statistically significant differences were observed in the remaining age group comparisons. Individuals demonstrated significant differences in their scores based on their education level. Specifically, TEI graduates demonstrated a statistically significant lower level of knowledge compared to both HEI graduates and MSc/PhD graduates. No statistically significant differences were observed in knowledge scores when comparing across places of residence, employment status, work experience, or the income of individuals.

### 3.3. The Use of the Science of Nutritional Genomics in Everyday Practice and Future Perspectives

**Figure 1:**
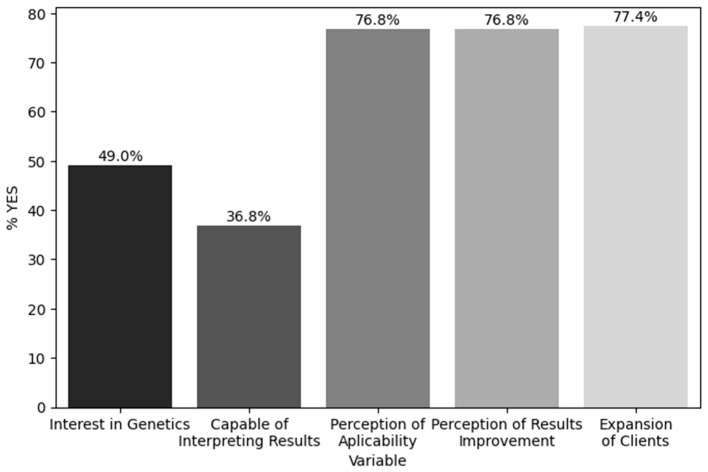
Previous interest in genetics and gene–diet interactions, perception of capability of interpreting Nutritional Genomics, perception of applicability of Nutritional Genomics in their clientele, perception of enhancement of their client’s goals, and of enhancement of client expansion by applying Nutritional Genomics results.

**Figure 2:**
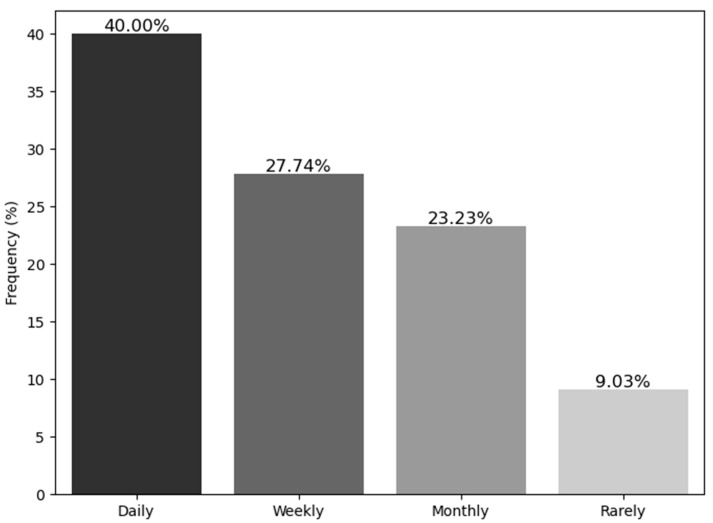
Frequency of updates on nutritional science.

The last part of the questionnaire focused on participants’ personal opinions on the science of Nutritional Genomics and the self-assessment of their current knowledge of the field, as well as its use in clinical practice and future perspectives. Figure 1 and Figure 2 depict the descriptive statistics of the answers in this third part of the questionnaire, while Table 4 presents the scores achieved based on the aforementioned answers. Interestingly, while 67.7% of the participants were informed on nutritional science updates at least once per week, only 49% of the participants reported previous interest in the science of genetics and gene–diet interactions. Out of these participants, 50% had been exposed to these sciences through additional educational courses or seminars, 25% through an MSc program, 19.7% through research work, and the remaining participants through the use of Direct-to-Consumer Genetic Testing in their private practices. Moreover, 52.3% and 37.4% of the respondents reported that their knowledge on these topics was low or intermediate, respectively, which aligns with the fact that 61.3% of the participants lacked confidence in their ability to interpret the results of a nutrigenetic test. Indeed, the higher the self-reported level of knowledge on the field, the higher the S-score. Despite the fact that the majority of the participants had no previous experience in the field of Nutritional Genomics, 76.8% of them believed that the application of this science to their clients would be feasible and that it could also support their clients in improving their results and in reaching their goals. Last but not least, 77.4% of the dietitians/nutritionists believed that specializing in this field would help them expand their clientele.

To explore the factors influencing the S-scores among this sample of Greek dietitians/nutritionists, a comprehensive linear regression analysis was performed (Table 5). The results highlighted significant associations between S-scores and several key variables: female sex, age group, education level, perceived confidence in interpreting genetic testing results, prior interest in genetics and gene–diet interactions, frequency of engagement with updates in nutritional science, and belief in the practical impact of genetic testing on client outcomes.

## 4. Discussion

This survey-based study explored the knowledge and perspectives of Greek dietitians and nutritionists regarding nutrigenetics, nutrigenomics, and genetic testing in practice. Our findings align with previous research in the United States, Canada, and the United Kingdom, where registered dietitians have consistently demonstrated limited knowledge and low confidence in applying Nutritional Genomics [15,16,17,18]. The mean correct response rate (57.7% ± 21.7%) suggests a moderate level of knowledge, comparable to studies in the United States, United Kingdom, and Australia (56%) but higher than those in India (42.3%) and Turkey (42%) [19,20,21]. These findings underscore the global need for enhanced genetic literacy among nutrition professionals.

Examining questionnaire responses, ~70% of the participants correctly identified the definitions of nutrigenetics (70.3%) and nutrigenomics (66.5%), yet only 21.3% knew the approximate number of human genes. While 78.7% recognized gene–disease associations, knowledge of specific genes (FTO and BDNF) was limited (34.2% and 6.5%, respectively). These results align with the study by Mathew et al. [19], where general genetic knowledge was adequate but the understanding of specific gene functions was poor. Despite this, 66.5% of participants demonstrated basic competency in interpreting nutrigenetic tests, consistent with similar findings [19]. Overall, some survey questions, such as the total number of human genes, required factual recall rather than applied competency in Nutritional Genomics. While these questions assess foundational knowledge, they may not fully capture practical understanding. Future questionnaire adaptations could include scenario-based or case-study questions to better evaluate real-world applications.

Historically, Nutritional Genomics education in Greek undergraduate dietetics programs was optional and included only in select curricula. Until recently, TEI and HEI programs did not offer nutrigenetics and nutrigenomics as mandatory courses, limiting exposure to these topics. However, recent curriculum reforms have incorporated them as core subjects in most programs. Since participants in this study completed their education before these changes, their moderate knowledge levels likely reflect the previous lack of structured training. These findings highlight the need for continuing education initiatives to bridge knowledge gaps among practicing dietitians and nutritionists.

Demographic factors significantly influenced knowledge levels. Women performed better than men (*p* = 0.046), particularly on the easier questions (*p* = 0.006), though the predominantly female sample (83.2%) limits broader conclusions. Age differences were evident, with younger professionals (25–35 years) scoring higher than those over 45 (*p* = 0.019), suggesting that recent graduates benefit from more updated curricula. Higher education levels correlated with better knowledge scores, with MSc and PhD holders outperforming TEI graduates (*p* < 0.001). These findings align with other studies showing that nutrigenomics education is primarily introduced at the postgraduate level [22,23].

Employment status and prior interest in genetics also influenced responses, whereas no significant differences were found based on work experience, place of residence, or income. Interestingly, participants with previous exposure to genetics scored significantly higher across all knowledge domains, emphasizing the importance of continuous learning. Despite knowledge limitations, 76.8% of participants believed Nutritional Genomics could benefit their clients and 77.4% expressed interest in specialization. However, 61.3% lacked confidence in interpreting genetic test results, mirroring trends in a 2018 Polish study, where 63% of dietitians desired further education [24]. Similarly, 88% of respondents in this study believed specialization in genetics would enhance their professional practice.

Regression analysis identified sex, age, education level, prior interest in genetics, frequency of engagement with scientific updates, and confidence in interpreting genetic test results as key predictors of knowledge scores. These findings reinforce that both academic background and professional engagement shape genomic literacy.

Given the increasing integration of Nutritional Genomics in personalized nutrition and preventive medicine, structured education in dietetics curricula and continuing professional training is essential [23]. A 2018 study reported that 94% of dietitians considered their knowledge insufficient, supporting the need for expanded nutrigenomics education [22]. Similarly, studies emphasize that genetic literacy is crucial for effectively incorporating genetic testing into dietetic practice [21,22,25].

To our knowledge, this is the first study in Greece to explore this topic, providing a foundation for further research into the evolving role of genetics in dietetic practice. However, there are several limitations. Although the sample size exceeded initial targets, it may not fully represent all Greek dietitians. The online questionnaire format allowed participants to potentially consult external sources, possibly inflating scores, whereas varying levels of prior knowledge may have influenced responses. The aim of the study, however, was to assess general knowledge in Nutritional Genomics rather than assess deep technical expertise. Additionally, the questionnaire, while based on the established literature, was not formally validated, though its high reliability (Cronbach’s α = 0.867) supports its consistency. Although this questionnaire was not externally validated prior to this study, its high Cronbach’s Alpha score supports its strong internal consistency and suitability for assessing knowledge in Nutritional Genomics. We recognize the existence of validated genetic knowledge surveys and acknowledge the value of cross-validation. Future research could further strengthen our tool’s robustness through examining test–retest reliability or comparisons with established instruments.

## 5. Conclusions

The field of Nutritional Genomics could lead dietitians and nutritionists into using new, tailor-made approaches in the field of nutrition, with regard to genetics and correlated diseases. Most of the current studies highlight the low level of knowledge of dietitians/nutritionists in this field, despite having a high perception of its benefits for their work and clients. The present study evaluated the level of knowledge of Greek dietitians/nutritionists in Nutritional Genomics. Most participants indicated a moderate level of knowledge in this field but expressed a high willingness to learn. The findings of this study suggest that both demographic characteristics as well as foundational education and continuous professional development are critical for enhancing genetic literacy in this field, suggesting the emerging need to incorporate nutrigenetics and nutrigenomics into dietetics curricula at universities. Furthermore, as nutrition has become an integral part of preventive medicine, it is essential for dietitians, nutritionists, and other HCPs to receive thorough education in this area [2].

## Figures and Tables

**Table 1:** Demographic characteristics of sample population.

Variable	Categories	*n* (%)
Sex	Male	26 (16.8%)
	Female	129 (83.2%)
Age group	<25	20 (12.9%)
	25–35	73 (47.1%)
	36–45	45 (29.0%)
	>46	17 (11.0%)
Education	TEI graduate	53 (34.2%)
	HEI graduate	44 (28.4%)
	MSc graduate	53 (34.2%)
	PhD graduate	5 (3.2%)
Work experience (years)	<5	67 (43.2%)
	5–10	39 (25.2%)
	11–20	41 (26.5%)
	>20	8 (5.2%)
Employment status	Currently unemployed	18 (11.6%)
	Currently employed	137 (88.4%)
Occupation type	Currently unemployed	18 (11.6%)
	Dietitian’s office	76 (49%)
	Home visits	26 (16.8%)
	Not working	18 (11.6%)
	Hospital/private clinic	11 (7.1%)
	Research	5 (3.2%)
	Pharmaceutical company	2 (1.3%)
	Gym	2 (1.3%)
	Education	1 (0.6%)
	Municipality	1 (0.6%)
	Journalism and social media	1 (0.6%)
	Other	12 (7.7%)
Place of residence	Village	9 (5.8%)
	Countryside	21 (13.5%)
	City (>20,000 residents)	125 (80.6%)
Income (€)	<500	30 (19.4%)
	500–1000	61 (39.4%)
	1000–2000	41 (26.5%)
	2000–3000	16 (10.3%)
	>3000	7 (4.5%)

Data are presented as *n* and % of all subcategories for each variable. TEI: Technological Educational Institute; HEI: Higher Educational Institution; MSc: Master of Science; PhD: Doctor of Philosophy.

**Table 2:** The participants’ knowledge, assessed using a 25-item scale on Nutritional Genomics. The questions are sorted in descending order based on the percentage of correct answers.

Questions/Declarations	Correct Answers (%)
8. Does each organism respond differently to food components?	83.2%
21. On what measurements are commercially available nutrigenetic tests based?	83.2%
12. Does the science of nutrigenetics aim to change our genetic profile?	80.6%
22. Following up on the previous question, how is the sampling conducted?	80.0%
13. Currently, have many genes responsible for various conditions been analyzed?	78.7%
23. How is Deoxyribonucleic Acid (DNA) isolated for conducting the test?	78.7%
24. Genetic polymorphism testing provides the necessary information for the scientist to create a personalized nutrition plan for the individual.	76.8%
20. Nutrigenetic testing is not relevant for athletes or young children at all.	75.5%
6. Nutrigenetics is the science that studies how the body responds to different nutrients based on its genetic background?	70.3%
25. With the help of nutrigenetic testing, is the dietitian able to provide recommendations on the amounts of caffeine, alcohol, and salt that the individual should consume?	66.5%
7. Nutrigenomics studies the role of food in how genes are expressed. In other words, this science explores how the function and expression of genes are influenced by nutrients.	66.5%
9. Does nutrigenetics study gene polymorphisms?	63.9%
4. Has knowledge of nutrigenetics and nutrigenomics facilitated a better understanding and potential practical application of molecular nutrition?	63.2%
18. Does a reliable nutrigenetic test provide information about diseases that the individual will definitely develop?	61.3%
5. Is nutrigenetics a type of diet?	60.0%
19. How often is it recommended to undergo a nutrigenetic test?	57.4%
17. Are there polymorphisms that appear to promote weight loss after adhering to dietary plans with specific macronutrient ratios (e.g., high-protein or low-fat diets)?	56.8%
10. Does a single nucleotide polymorphism (SNP) result from the substitution of a base in an allele and is the most common cause of genetic diversity?	41.9%
3. Our genome differs by 0.1%.	41.9%
1. Has the decoding of the human genome already been completed?	34.2%
15. Is the most common gene associated with the onset of Alzheimer’s disease called Apolipoprotein E (APOE)?	34.2%
14. Is the Fat mass and obesity-associated (FTO) gene directly related to hypertension?	29.7%
11. Do SNPs change during our lifetime?	26.5%
2. The human genome consists of approximately 2.9 billion nucleotides/20,000 coding genes?	21.3%
16. Has the brain-derived neurotrophic factor (BDNF) gene been associated with reduced food intake in children?	6.5%

**Table 3:** Association between participants’ demographic characteristics and knowledge scores.

Variable	S-Score	*p*-Value	E-Score	*p*-Value	H-Score	*p*-Value
Sex						
Female	14.84 ± 5.19	0.046	9.15 ± 2.81	0.006	5.69 ± 2.75	0.157
Male	12.15 ± 6.17		7.38 ± 3.62		4.77 ± 3.00	
Age group						
<25	14.95 ± 4.70	0.031	8.80 ± 2.82	0.050	6.15 ± 2.25	0.040
25–35	15.10 ± 5.09 *		9.26 ± 2.81 *		5.84 ± 2.68 *	
36–45	14.33 ± 5.71		8.91 ± 3.09		5.42 ± 2.97	
>45	10.82 ± 5.96 *		7.00 ± 3.48 *		3.82 ± 3.0 *	
Education						
TEI	12.19 ± 5.48 *⤉	0.0003	7.72 ± 3.27 *⤉	0.001	4.47 ± 2.62 *⤉	0.001
HEI	16.27 ± 4.05 *		9.70 ± 2.16 *		6.57 ± 2.28 *	
MSc	15.36 ± 5.66 ⤉		9.47 ± 3.02 ⤉		5.89 ± 3.04 ⤉	
PhD	10.80 ± 4.60		6.80 ± 2.78		4.00 ± 2.55	
Place of residence						
Village	12.89 ± 4.99	0.479	8.78 ± 2.95	0.861	4.11 ± 2.52	0.159
Small city	13.52 ± 5.65		8.52 ± 2.99		5.00 ± 2.93	
City (>20,000 residents)	14.64 ± 5.44		8.91 ± 3.05		5.73 ± 2.78	
Employment status						
Currently employed	14.56 ± 5.41	0.296	9.00 ± 3.03	0.083	5.56 ± 2.78	0.765
Currently unemployed	13.06 ± 5.63		7.72 ± 2.78		5.33 ± 3.05	
Work experience (years)						
<5	14.49 ± 5.54	0.693	8.73 ± 3.07	0.817	5.76 ± 2.83	0.435
5–10	15.05 ± 5.26		9.26 ± 2.88		5.79 ± 2.80	
11–20	13.83 ± 5.84		8.71 ± 3.33		5.12 ± 2.89	
>20	13.13 ± 3.14		8.63 ± 1.30		4.50 ± 2.00	
Income (€)						
<500	14.87 ± 5.08	0.703	8.87 ± 2.75	0.735	6.00 ± 2.83	0.577
500–1000	14.89 ± 4.78		9.15 ± 2.66		5.74 ± 2.56	
1001–2000	13.37 ± 5.86		8.34 ± 3.24		5.02 ± 2.96	
2001–3000	14.25 ± 7.06		8.75 ± 4.22		5.50 ± 2.99	
>3000	14.29 ± 6.34		9.43 ± 3.05		4.86 ± 3.58	

Values are presented as mean ± standard deviation. *t*-test for independent samples, one-way ANOVA, and post hoc Bonferroni tests were used. *⤉ *p*-values for pairwise comparisons ≤ 0.05. TEI: Technological Educational Institute; HEI: Higher Educational Institution; MSc: Master of Science; PhD: Doctor of Philosophy.

**Table 4:** Knowledge scores and participants’ perspectives on adequacy of their knowledge and practical relevance of this field.

Variable	S-Score	*p*-Value	E-Score	*p*-Value	H-Score	*p*-Value
Interest in genetics						
No	4.25 ± 2.66	0.001	7.41 ± 3.07	0.001	4.25 ± 2.66	0.041
Yes	6.87 ± 2.29		10.36 ± 2.10		6.87 ± 2.29	
Capable of interpreting results						
No	12.54 ± 5.26	0.026	7.85 ± 3.04	0.003	4.69 ± 2.64	0.240
Yes	17.56 ± 4.12		10.58 ± 2.06		6.98 ± 2.47	
Perception of applicability						
No	11.39 ± 6.40	0.004	7.17 ± 3.66	0.002	4.22 ± 3.01	0.077
Yes	15.29 ± 4.78		9.36 ± 2.61		5.93 ± 2.63	
Perception of results improvement						
No	10.69 ± 5.62	0.257	6.78 ± 3.41	0.012	3.92 ± 2.58	0.880
Yes	15.5 ± 4.87		9.48 ± 2.59		6.03 ± 2.69	
Expansion of clients						
No	12.86 ± 6.51	0.016	7.83 ± 3.80	0.001	5.03 ± 3.15	0.240
Yes	14.83 ± 5.02		9.15 ± 2.70		5.68 ± 2.69	
Self-reported level of knowledge						
Low	12.01 ± 5.20 *	<0.001	7.60 ± 2.97 *	<0.001	4.41 ± 2.69 *	<0.001
Intermediate	16.36 ± 4.5 *		10.02 ± 2.58 *		6.34 ± 2.28 *	
Good	18.85 ± 3.63 *		10.69 ± 1.75 *		8.15 ± 2.34 *	
Very good	21 ± 1 *		12.00 ± 1 *		9 ± 1 *	
Frequency of updates on nutritional science						
Rarely	8.86 ± 5.99 *	<0.001	5.79 ± 3.73 *	<0.001	3.07 ± 2.46 *	<0.001
Monthly	13 ± 4.55		8.33 ± 2.40 *		4.67 ± 2.59 ⤉	
Weekly	15.53 ± 5.4 *		9.21 ± 3.03 *		6.33 ± 2.83 *⤉	
Daily	15.65 ± 4.92 *		9.6 ± 2.73 *		6.05 ± 2.58 *	

Values are presented as mean ± standard deviation. *t*-test for independent samples, one-way ANOVA and post hoc Bonferroni tests were used. *⤉ *p*-values for pairwise comparisons ≤ 0.05.

**Table 5:** Assessment of S-score predictors via backward linear regression model.

	Beta	SE	*p*-Value
Sex (female vs. male)	1.372	0.846	1.07 × 10^−1^
Age group (Ref. <25 years old)			
25–35	0.829	1.037	4.26 × 10^−1^
36–45	0.316	1.170	7.87 × 10^−1^
>45	−2.566	1.376	6.44 × 10^−2^
Education (Ref. TEI)			
HEI	2.713	0.828	1.33 × 10^−3^
MSc	2.118	0.757	5.92 × 10^−3^
PhD	2.492	1.876	1.87 × 10^−1^
Interest in genetics (yes vs. no)	3.002	0.708	4.14 × 10^−5^
Capable of interpreting results (yes vs. no)	1.986	0.752	9.07 × 10^−3^
Frequency of updates on nutritional science (Ref. rarely)			
Monthly	2.245	1.244	7.32 × 10^−2^
Weekly	4.203	1.188	5.50 × 10^−4^
Daily	3.401	1.179	4.55 × 10^−3^
Perception of results improvement (yes vs. no)	2.859	0.761	2.52 × 10^−4^

Ref.: reference; TEI: Technological Educational Institute; HEI: Higher Educational Institution; MSc: Master of Science; PhD: Doctor of Philosophy.

## Data Availability

The dataset is available on request from the authors. The data are not publicly available due to ethical reasons.

## Supplementary Materials

The following supporting information can be downloaded at: https://www.mdpi.com/article/10.3390/nu17071107/s1. Questions and answers are included in the “Questionnaire on Nutritional Genomics—Part 2: Knowledge Assessment”.

## Abbreviations

The following abbreviations are used in this manuscript:

SNPs	Single nucleotide polymorphisms
HCPs	Healthcare professionals
DTCGT	Direct-to-Consumer Genetic Testing
GDPR	General Data Protection Regulation
FDA	Food and Drug Administration
RDNs	Registered dietitians and nutritionists
ANOVA	Analysis of variance
TEI	Technological Educational Institute
HEI	Higher Education Institution
MSc	Master of Science
PhD	Doctor of Philosophy
