# Supplementary material for: Assessing the Knowledge and Beliefs of Greek Dietitians and Nutritionists on Nutritional Genomics: A Survey-Based Study
Source: Nutrients. 2025 Mar 21;17(7):1107. doi: 10.3390/nu17071107 (PMC11990335; doi:10.3390/nu17071107)
Supplement: Supplementary file 1 [file nutrients-17-01107-s001.zip › nutrients-3487987-supplementary.pdf]

Questionnaire on Nutritional Genomics - Part 2: Knowledge Assessment

1. **Has the decoding of the human genome already been completed?**

   a. Yes
   b. No
   c. I don’t know

2. **Does the human genome consist of approximately 2,9 billion nucleotides and 20,000 genes?**

   a. Yes
   b. No
   c. I don’t know

3. **Our genome differs by 0.1%.**

   a. Yes
   b. No
   c. I don’t know

4. **Has knowledge of nutrigenetics and nutrigenomics facilitated a better understanding and potential practical application of molecular nutrition?**

   a. Yes
   b. No
   c. I don’t know

5. **Is nutrigenetics a type of diet?**

   a. Yes
   b. No
   c. I don’t know

6. **Nutrigenetics is the science that studies how the body responds to different nutrients based on its genetic background.**

   a. Yes
   b. No
   c. I don’t know

7. **Nutrigenomics studies the role of food in how genes are expressed. In other words, this science explores how the function and expression of genes are influenced by nutrients.**

   a. Yes
   b. No
   c. I don’t know

8. **Does each organism respond differently to food components?**

a. Yes
b. No
c. I don’t know

9. Does nutrigenetics study gene polymorphisms?

a. Yes
b. No
c. I don’t know

10. Does a single nucleotide polymorphism (SNP) result from the substitution of one base in an allele and is the most common cause of genetic diversity?

a. Yes
b. No
c. I don’t know

11. Do SNPs change throughout our lifetime?

a. Yes
b. No
c. I don’t know

12. Does the science of nutrigenetics aim to change our genetic profile?

a. Yes
b. No
c. I don’t know

13. Currently, have many genes responsible for various conditions been analysed?

a. Yes
b. No
c. I don’t know

14. Is the Fat mass and obesity-associated (FTO) gene directly related to hypertension?

a. Yes
b. No
c. I don’t know

15. Is the most common gene associated with the onset of Alzheimer's disease called Apolipoprotein E (APOE)?

a. Yes
b. No
c. I don’t know

16. Has the Brain-Derived Neurotrophic Factor (BDNF) gene been associated with reduced food intake in children?

a. Yes
b. No
c. I don’t know

17. Are there polymorphisms that appear to promote weight loss after adhering to dietary plans with specific macronutrient ratios (e.g., high-protein or low-fat diets)?

a. Yes
b. No
c. I don’t know

18. Does a reliable nutrigenetic test provide definitive information about diseases an individual will develop?

a. Yes
b. No
c. I don’t know

19. How often is it recommended to undergo a nutrigenetic test?

a. Every 5 years
b. Every 10 years
c. As many times as the individual wants
d. Once in a lifetime
e. I don’t know

20. Nutrigenetic testing is not relevant for athletes or young children at all.

a. Yes
b. No
c. I don’t know

21. On what measurements are commercially available nutrigenetic tests based?

a. Fat
b. Bone density
c. Muscle mass
d. Genetic material
e. I don’t know

22. Following up on the previous question, how is the sampling conducted?

a. Urine test
b. Bioelectrical impedance analysis
c. Saliva sample

d. Skin swab
e. I don’t know

23. How is Deoxyribonucleic Acid (DNA) isolated for conducting the test?

a. Needle extraction
b. Cotton swab
c. Ultrasound
d. CT scan
e. I don’t know

24. Genetic polymorphism testing provides the necessary information for the scientist to create a personalised nutrition plan for the individual.

a. Yes
b. No
c. I don’t know

25. With the help of nutrigenetic testing, is the dietitian able to provide recommendations on the amounts of caffeine, alcohol, and salt that the individual should consume?

a. Yes
b. No
c. I don’t know
